# Research progress on the mechanism of orexin in pain regulation in different brain regions

**DOI:** 10.1515/biol-2021-0001

**Published:** 2021-01-20

**Authors:** Xianhui Kang, Hongli Tang, Yao Liu, Yan Yuan, Mi Wang

**Affiliations:** Department of Anesthesiology, The First Affiliated Hospital, Zhejiang University School of Medicine, Hangzhou, Zhejiang Province 310003, China; Department of Anesthesiology, The First Affiliated Hospital of Wenzhou Medical University, Wenzhou City, Zhejiang Province 325000, China; Department of Pain Management, Jiangnan University, No.1000 Hefeng Road, Binhu District, Wuxi, Jiangsu Province 214000, People’s Republic of China; Department of Anesthesiology, The Affiliated Hospital of Xuzhou Medical University, No. 84 Huaihai West Road, Quanshan District, Xuzhou, Jiangsu Province 221002, People’s Republic of China

**Keywords:** orexins, orexin A, pain regulation, orexin receptors, acute pain

## Abstract

Orexin is a neuropeptide that is primarily synthesized and secreted by the lateral hypothalamus (LH) and includes two substances derived from the same precursor (orexin A [OXA] and orexin B [OXB]). Studies have shown that orexin is not only involved in the regulation of eating, the sleep–wake cycle, and energy metabolism, but also closely associated with various physiological functions, such as cardiovascular control, reproduction, stress, reward, addiction, and the modulation of pain transmission. At present, studies that have been performed both domestically and abroad have confirmed that orexin and its receptors are closely associated with pain regulation. In this article, the research progress on acute pain regulation involving orexin is reviewed.

## 1 Introduction

Orexins, which are also known as hypocretins, are named according to their strong orexigenic effects and include orexin A (OXA) and orexin B (OXB), both of which are derived from the same precursor protein (pro-orexin). Their receptors are G protein-coupled receptors and include orexin receptor 1 (OX1R) and orexin receptor 2 (OX2R). OXA has almost the same affinity for OX1R and OX2R, whereas OXB has a tenfold higher affinity for OX2R than OX1R [1]. Northern blot analyses of the RNA of adult rats have shown that in the brain, orexins and their receptors are only expressed in the adjacent lateral and posterior regions of the hypothalamus, which are associated with feeding behavior and energy regulation [2]. Although the number of hypothalamic orexin neurons is extremely limited [3], they are able to project to many pain-related brain regions, including the thalamus, limbic system, dorsal raphe nucleus (DR), locus coeruleus (LC), periaqueductal gray (PAG) matter, dorsal hippocampus, reticular formation, and trigeminal caudate nucleus [4]. Numerous studies have shown that in various animal models of inflammatory pain induced by formalin, capsaicin, and carrageenan and in chronic neuropathic pain animal models, the injection of exogenous orexin into the spinal cord and supraspinal sites that are associated with the descending pain regulatory circuits can significantly reduce nociceptive responses. In this article, the current research status of the involvement of orexin in pain regulation in different brain regions is reviewed, with a goal of providing additional references to further clarify the analgesic mechanism of orexin.

## 2 Spinal cord

The spinal cord is the primary center of pain modulation, and the superficial spinal dorsal horn (DH) (laminae I and II; lamina II is also known as the substantia gelatinosa) is an important site for the transmission and integration of peripheral nociceptive signals [5]. As the principal fibers transmitting mechanical, temperature, and noxious chemical signals from the periphery to the DH, Aδ and C fibers form synapses with DH neurons (especially with spinal glial cells), and these synapses are the key sites for modulating pain signals. Hypothalamic orexin neurons send long-axis projections throughout DH laminae I–II, and OXA content is most abundant in these two laminae [6]. Rezaee et al. [7] have also found that the intrathecal administration of OXA can significantly reduce behavioral responses to thermal and mechanical pain in rats, indicating a potential role of OXA in sensation and pain modulation.

Furthermore, Jeon et al. [6] found that the application of OXA can reduce the amplitude of excitatory postsynaptic currents that are elicited by the electrical stimulation of Aδ or C fibers via the significant inhibition of OX1R-mediated excitatory synaptic transmission [6], which has previously been demonstrated in many animal models of neuropathic pain caused by inflammatory pain and peripheral nerve injury. Subsequently, the same researcher performed a study on long-term depression (LTD) and orexin receptor-mediated DH excitatory synaptic transmission by using the whole-cell patch-clamp technique, and the data showed that OX1R and OX2R antagonists were able to reduce the amplitude of LTD whereas antagonistic effect of OX2R was more significant [8]. This finding indicates that OXA can exert pain modulatory effects at the spinal cord level via different receptors. Studies have also shown that OX2R is densely expressed in the superficial spinal DH in rats [9]. Wang recorded changes in the amplitudes and frequencies of excitatory and inhibitory postsynaptic currents via intrathecal injections of OXB and OXA, as well as via the use of OX1R and OX2R antagonists, in adult rats. The results indicated that OXB can also play a role in modulating pain at the spinal cord level and that the analgesic effects of both OXB and OXA occurred via the activation of OX2R in spinal lamina II [10].

## 3 PAG matter of the midbrain and dorsal raphe nucleus

The ventrolateral periaqueductal gray (vlPAG) matter of the midbrain is an important site for modulating pain and mood [11] that receives projections from hypothalamic orexin neurons. Studies have shown that after OXA binds to OX1R in the postsynaptic membrane of the vlPAG matter, an endocannabinoid (2-arachidonoylglycerol [2-AG]) is produced via the phospholipase C (PLC)–diacylglycerol lipase (DAGL) pathway [12]. Subsequently, 2-AG acts in a retrograde manner to activate the cannabinoid 1 receptor (CB1R) at the presynaptic membrane, which enables the activation of the vlPAG excitatory neurons that project to the rostral ventromedial medulla (RVM) by inhibiting the abundant GABAergic interneurons in the vlPAG matter. Additionally, the descending pain inhibitory pathway (constituted by the vlPAG-RVM-DH circuit) exerts analgesic effects [13,14]. Studies have shown that injections of OX2R antagonists into the vlPAG matter can block the analgesic effects produced by the injection of carbachol into the lateral hypothalamus (LH) in the tail-flick test, indicating that OX2R in the vlPAG matter is also involved in the modulation of pain and that the analgesic effect of OX2R is not dependent on CB1R [15]. Experiments by Okumura [16] have shown that both CB1R and CB2R antagonists can effectively block the antinociceptive actions of orexin against colonic distention, suggesting that CB2R may also mediate orexin-induced visceral analgesia and that the site of action of CB2R antagonists may be in the brain. However, the specific pathways in which CB2R is involved remain to be further investigated.

The DR, as part of the vlPAG, contains an abundant number of serotonergic neurons and is a vital part of the endogenous pain modulation system. Moreover, we cannot deny the fact that the orexin system is relevant in pain perception, wakefulness, and integration, with its activation linked to circadian periodicity [17]. Antonietta analyzed clinical symptoms and laboratory results in their study and concluded that sleep disorders, such as nocturnal awakenings, insomnia, parasomnias, and food selectivity in autism spectrum disorder (ASD) children can be explained by increased cerebral metabolism and the hyperfunctioning of the autonomic nervous system, which is sustained by high OXA levels [18]. Furthermore, numerous cross-sectional studies have demonstrated a high degree of comorbidity between pain and sleep impairments [19]. Serotonergic neurons, which are well-known as being a central component in migraine attacks, receive an excitatory input from hypothalamic orexin neurons and can reciprocally inhibit orexin neurons through the serotonin 1A receptor. In some cases, multidirectional excitatory connections are present among several brain nuclei (such as PAG and DR), which results in even further complications to the overall mechanism [20]. Researchers have hypothesized that if this system is dysregulated or disrupted, it may facilitate the pathophysiological mechanisms involved in migraines; in addition, the system may simultaneously produce an alteration of the sleep–wake rhythm, thus causing sleep disorders [21].

## 4 Hippocampus

The hippocampus is not solely a site for regulating emotions, controlling learning and memory, and participating in stress responses; recent studies have shown that the hippocampus is also involved in the regulation of nociception [22]. Partial hippocampal resection has been used for the treatment of chronic pain. Orexinergic fibers project from the LH to the dorsal Cornu Ammonis 1 (CA1) area of the hippocampus, and orexin receptors are distributed in different brain regions associated with memory, including the hippocampus. The injections of OX1R antagonists into the CA1 region of the hippocampus can inhibit the analgesic effect of the LH in a formalin inflammatory pain model in a dose-dependent manner, indicating the existence of a neural pathway from the LH to the CA1 region that modulates pain [23]. Kooshki injected OXA or OX1R antagonists into the CA1 region and observed that the pain responses in capsaicin-treated rats were significantly reduced; furthermore, learning and memory losses were also reduced in these rats [24]. The orexin system is involved in the stress response mediated via the hypothalamic–pituitary axis (HPA). Results from Bahramzadeh et al. [25] revealed that the application of OX1R and OX2R antagonists in the hippocampus can prevent anxiety, immobility, and escape behavior caused by acute stress in rats in the elevated plus maze and in open field tests, indicating that OX1R antagonists can improve the adaptation of rats to stress. However, the specific mechanism of this effect is not yet clear.

## 5 Rostral ventromedial medulla

The RVM is an important aspect of the descending pain inhibitory system. In the formalin test, microinjections of OXA into the RVM can reduce the pain response, especially in the second phase of formalin-induced inflammatory pain. Furthermore, this analgesic effect can be blocked by a selective OX1R antagonist, indicating that the analgesic effect of OXA is at least partially achieved through OX1R in the RVM [26]. Some researchers have suggested that the first phase of the formalin test is due to the toxic and destructive effects of formalin on the tissues surrounding the injection site and due to the repetitive stimulation of C-fiber nociceptors. In addition, the second phase is due to the central sensitization of the spinal cord caused by the inflammatory response to tissue injury via the release of inflammatory factors and the long-term response of DH neurons to repeated C-fiber inputs [27]. Interestingly, Haghparast observed that the effects of both OX1R and OX2R antagonists were more prominent during the first phase of LH-induced antinociception that occurred during formalin-induced orofacial pain. Therefore, it is speculated that two different nerves are responsible for pain transduction in facial and limb areas [28]. Apart from the descending pain inhibition pathway constituted by the vlPAG-RVM-DH circuit, the question of whether RVM plays a role in other pathways remains to be extensively studied.

## 6 Locus coeruleus

The LC is an aggregated cluster of noradrenergic neurons in the brainstem, and orexinergic neurons send extensive projections to the LC region; additionally, OX1R is highly expressed in LC neurons. In a formalin experiment, investigators found that microinjections of OXA into the LC can play a role in analgesia, whereas pretreatment with a CB1R antagonist inhibited this effect, thus leading to hyperalgesia [29]. Endorphins are morphine-like transmitters in the central nervous system, and OX1R antagonists in the LC can effectively block the analgesic effects of microinjected endorphins, thus suggesting that the analgesic effects of endorphins in the LC may be mediated by the orexinergic system [30]. Experiments using brain sections of rats with desensitized μ-opioid receptors (MORs) (caused by endorphin exposure) revealed that OXA increased the maximum extent and rate of MOR desensitization and that pretreatment with a protein kinase C inhibitor can significantly inhibit the effect of OXA on the degree of desensitization. However, it had no significant effect on the rate of desensitization, which led to the speculation that OXA affects these two processes via two cellular mechanisms (PKC-dependent and PKC-independent mechanisms) [31]. Abdollahi injected morphine into the LC of rats and used OX1R antagonists to prolong the duration of morphine tolerance in these rats [32]. This led to the speculation, that OXA accelerates the development of morphine tolerance [32]. Based on the previously described studies, it can be speculated that the increased orexin neuronal activity plays an important role in the development of drug-related adverse effects, such as resistance and dependence, during long-term exposure to opioids. Moreover, we cannot neglect to mention that strong excitatory inputs from the lateral paragigantocellularis (LPGi) nuclei have been shown to affect LC neuronal responsiveness during the occurrence of opiate dependence and tolerance [33].

## 7 Ventral tegmental area (VTA) and nucleus accumbens (NAC)

The VTA comprises a group of neurons in the midbrain or near the midline and is the root of the dopaminergic cell bodies. Orexin can directly stimulate dopaminergic and nondopaminergic neurons via postsynaptic effects. The NAC is a collection of neurons that form the main part of the ventral striatum. Experimenters injected OX1R and OX2R antagonists into the VTA or NAC and found that they could inhibit the analgesic effects of carbachol injected into the LH. Therefore, they speculated that the pathway from the LH to the VTA and NAC plays an important role in pain modulation [34,35]. The study by Azhdari-Zarmehri found that the activation of orexin receptors in the VTA could increase dopamine release via neurons projecting to the NAC [36]. The findings of Okumura et al. indicated that levodopa induces an antinociceptive action against colonic distention by activating D2 dopamine receptors and the orexinergic system in the rat brain [37]. In addition, the results of another study indicated that D1 receptors in the NAC can also mediate the analgesic effect of OXA in the VTA (to some extent) and were involved in the regulation of acute nociceptive responses in rats [38].

## 8 Conclusion and future directions

**Figure 1:**
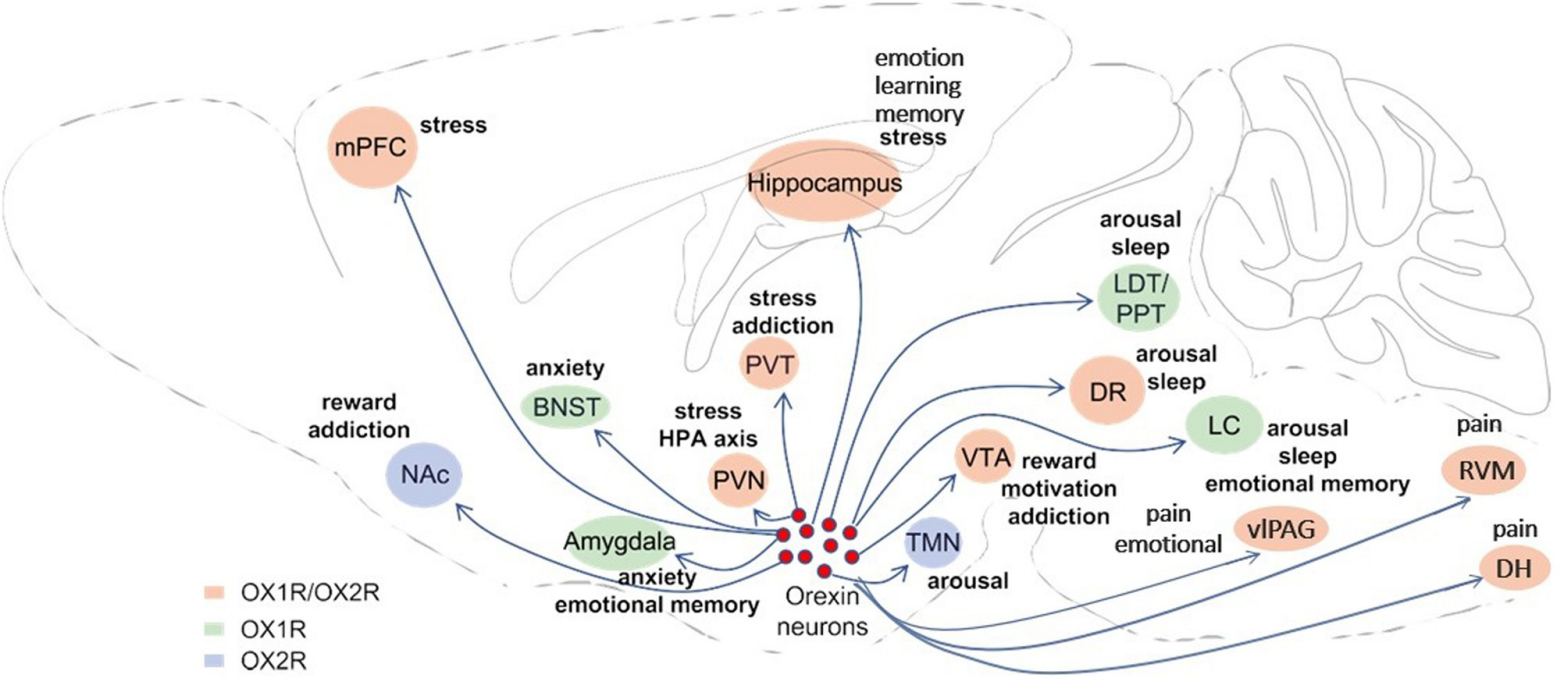
Efferent projections of orexin/hypocretin neurons, the distribution of orexin receptors, and the associated behavioral functions in postsynaptic target regions. Orexin neurons project widely throughout the brain onto target regions which include the spinal dorsal horn (DH), ventrolateral periaqueductal gray (vlPAG), dorsal raphe (DR), hippocampus, rostral ventromedial medulla (RVM), locus coeruleus (LC), ventral tegmental area (VTA), nucleus accumbens (NAC), the laterodorsal tegmental nucleus and the pedunculopontine tegmental nucleus (LDT/PPT), tuberomammillary nucleus (TMN), hypothalamic paraventricular nucleus (PVN), paraventricular nucleus of the thalamus (PVT), bed nucleus of the stria terminalis (BNST), amygdala, and the medial prefrontal cortex (mPFC). Each of these target regions is involved in the regulation of diverse behavioral and physiological functions.

Our work attempts to distinguish various brain structures to understand the multifaceted contribution of orexin neurons in endogenous pain regulation (Table 1). Taken together, the evidence indicates that orexin neurons are involved in the regulation of nociception via widespread projections to different parts of the central nervous system. At present, the research of the analgesic effects of orexins is mainly focused on animal experiments, and supporting evidence from clinical trials is mostly related to the treatment of migraines or cluster headaches, with these studies being rare. Studies have suggested that the analgesic effect of OXA is more certain, whereas OXB has a weaker modulatory effect on pain, and the effects of OX1R and OX2R vary greatly in different models. At present, analgesic drugs mainly include opioids and nonsteroidal anti-inflammatory drugs (NSAIDs), with opioids having side effects that include immunosuppression, constipation, nausea, vomiting, and might result in development of addiction caused by long-term use. Although specific cyclooxygenase inhibitors are continuously introduced, NSAIDs present both a slow onset of action and limited analgesic effects and result in adverse reactions in the gastrointestinal tract and in the cardiovascular and nervous systems. To date, orexin preparations have been used in the clinical treatment of sleep disorders, and the in-depth study of the analgesic mechanism of orexin provides a direction for the development of new pain medications. Notably, orexin peptides have been extensively shown to be involved in the regulation of various physiological functions not limited to pain modulation (Figure 1). Therefore, future researchers who are interested in the design of novel drugs that target the orexin system should focus on how these drugs act on specific parts of the brain and on the specific cytoarchitecture and distinct neurochemical characteristics of these drugs, in order for these drugs to reduce adverse reactions.

**Table 1:** Summary of the studies investigating the role of orexin system in pain and nociception modulation

Regions	Methods	Endogenous Neuromodulators	Receptors	Comorbidities	Ref.
DH	Electrical stimulation of Aδ- or C-primary afferent fibers	OrexinA	OX1R		[6]
	Thermal (hot-plate, tail-flick, paw-withdrawal), mechanical (tail-pressure), chemical (formalin, capsaicin, and abdominal stretch)	OrexinA, OrexinB	ND		[7]
	Electrical stimulation	ND	OX1R, OX2R		[8]
	Patch-clamp whole-cell technique	OrexinB	OX2R		[10]
vlPAG, DRN	Patch-clamp whole-cell recording	OrexinA	OX1R, OX2R, CB1R		[12]
	Mouse hot-plate test	OrexinA	OX1R, OX2R, CB1R		[13]
	Rat tail-flick test	ND	OX1R, CB1R		[14]
	Rat tail-flick test	ND	OX2R		[15]
	Rat abdominal constriction test	OrexinA	CB1R, CB2R		[16]
	Case report	ND	ND	Autism spectrum disorder (ASD) subjects may present a dysregulation in orexinergic neurotransmission	[18]
	Inference	ND	ND	Sleep-pain coregulation by orexin system	[20]
	Inference	ND	Serotonin 1 A receptor	In migraine, a dysfunction of orexinergic projections interfering with serotonergic regulation may cause parasomnias	[21]
Hippocampus	Rat formalin test	ND	OX1R		[23]
	Rat intra-lip injection of capsaicin-induced orofacial pain test	OrexinA	OX1R	Learning and memory loss	[24]
	Rat acute (two mild electric shocks, 5.5 mA) and chronic stresses (10 days of restraint, 6 h daily)	ND	OX1R, OX2R	Impairment of social novelty and anxiety behavior	[25]
RVM	Rat formalin test	OrexinA	OX1R		[26]
	Rat formalin-induced orofacial pain test	ND	OX1R, OX2R		[28]
LC	Rat formalin test	OrexinA	OX1R, CB1R		[29]
	Rat tail-flick test	ND	OX1R	Analgesic tolerance	[30]
	Patch-clamp whole-cell recording	OrexinA	OX1R	μ-Opioid receptor desensitization	[31]
	Vivo extracellular single unit recording	ND	OX1R	Morphine tolerance.	[32]
VTA	Rat formalin test	ND	OX2R		[34]
	Rat tail-flick test	ND	OX1R, OX2R		[35]
	Rat abdominal constriction test	ND	OX1R, D2 dopamine receptor		[37]
	Rat tail-flick test	OrexinA	D1/D2 dopamine receptor		[38]
